# Molecular and Biosafety Perspectives of Bacterial Self-Healing Concrete: From Sporulation and Biomineralization to Public Health Implications

**DOI:** 10.3390/ma19173661

**Published:** 2026-08-28

**Authors:** Cecilia Manrique-Sam, Ronel Rivas-Torres, Alejandro Miranda-Pinto, Johany Cecilia Sanchez Guillen, Sandra Apaza-Tosocahua, Fernando Farfán-Delgado

**Affiliations:** Faculty of Human Medicine, Universidad Católica de Santa María, Arequipa 04000, Peru; 75366881@estudiante.ucsm.edu.pe (R.R.-T.); amiranda@ucsm.edu.pe (A.M.-P.); jsanchezg@ucsm.edu.pe (J.C.S.G.); slapaza@ucsm.edu.pe (S.A.-T.); mfarfan@ucsm.edu.pe (F.F.-D.)

**Keywords:** bacterial self-healing concrete, microbially induced calcium carbonate precipitation, biomineralization, sporulation, biofilms, encapsulation, biosafety

## Abstract

Bacterial self-healing concrete has emerged as a bio-based strategy to enhance the durability of cementitious materials and reduce the environmental impact associated with premature infrastructure deterioration. Its functional principle relies on microbially induced calcium carbonate precipitation (MICP), through which bacterial metabolism promotes CaCO_3_ deposition within cracks. However, self-healing efficiency cannot be explained solely by mineral precipitation capacity. Concrete is a restrictive microbial environment characterized by alkalinity, desiccation, osmotic stress, nutrient limitation and physical confinement. Therefore, effective crack sealing requires a coordinated sequence involving bacterial survival, sporulation, germination, metabolic reactivation, biofilm-associated mineral nucleation and localized biomineralization. This integrative narrative review synthesizes mechanistic, material and biosafety evidence on bacterial self-healing concrete, focusing on spore-forming bacteria such as *Bacillus subtilis* and related taxa, including *Paenibacillus*. The evidence indicates that stress tolerance, germination signaling, calcium handling, biofilm establishment and stability, and encapsulation-mediated microenvironmental control are key determinants of performance, but remain insufficiently integrated into materials-oriented studies. Large-scale implementation also requires preventive assessment of strain persistence, genetic stability, horizontal gene transfer, environmental microbiome interactions and life-cycle exposure scenarios. Bacterial self-healing concrete should therefore be understood as a living or bioactive material system whose responsible development depends on the integration of microbiology, molecular biology, materials science, civil engineering, environmental risk assessment, occupational health, and public health.

## 1. Introduction

The environmental burden of the construction sector has intensified the search for cementitious materials with improved durability, reduced maintenance requirements and lower life-cycle impacts. Buildings and construction remain major contributors to global energy use and CO_2_ emissions, while cement and concrete production continue to face substantial challenges in the transition towards low-carbon infrastructure [1,2]. In this context, extending the service life of concrete structures is not only an engineering priority, but also a sustainability strategy, since crack formation accelerates water ingress, reinforcement corrosion, loss of mechanical performance and premature repair or replacement of infrastructure [3,4,5].

Bacterial self-healing concrete has emerged as a bio-based approach aimed at promoting autonomous crack sealing through microbially induced calcium carbonate precipitation (MICP). In this process, bacterial metabolism modifies the local chemical microenvironment and favors the precipitation of calcium carbonate within microfissures, thereby reducing permeability and contributing to mechanical recovery [3,4,5,6,7]. Spore-forming bacteria, particularly species of the genus *Bacillus*, have received special attention because endospore formation may allow long-term persistence during mixing, curing and exposure to the highly restrictive conditions of cementitious matrices [5,7]. Other spore-forming taxa, including *Paenibacillus*, have also been considered of biotechnological interest, although their comparative performance under concrete-relevant conditions remains less extensively characterized.

Despite these advances, the efficacy of bacterial self-healing concrete cannot be explained solely by the amount of calcium carbonate produced. Concrete is not a permissive microbial habitat: high alkalinity, osmotic stress, progressive desiccation, nutrient limitation and physical confinement can compromise bacterial viability and delay metabolic reactivation [8,9]. Therefore, successful self-healing requires a coordinated biological sequence involving survival under stress, sporulation or latency, germination after crack formation, metabolic reactivation and subsequent biomineralization. In this sequence, molecular mechanisms such as general stress responses, endospore differentiation, germinant receptor activation and cortex degradation become functional determinants of material performance rather than merely microbiological phenomena [10,11,12].

A second limitation in the field is that MICP is frequently described as a physicochemical precipitation process, while the biological regulation of biomineralization remains less integrated into material-level analyses. Evidence from *Bacillus subtilis* and related spore-forming bacteria indicates that calcium carbonate precipitation can involve specific genetic determinants, calcium transport systems, metabolic regulation and biofilm-associated mineral nucleation [13,14,15,16,17]. Biofilms may provide spatially organized scaffolds for mineral deposition, whereas encapsulation systems can protect spores during concrete preparation and modulate the availability of water, oxygen and nutrients after cracking [16,17,18,19]. These processes suggest that the design of bacterial self-healing concrete should integrate microbiological viability, molecular regulation, encapsulant architecture and cementitious compatibility.

As bacterial self-healing systems move from laboratory-scale formulations towards possible field implementation, their classification as living or bioactive construction materials also raises preventive questions related to environmental biosafety, occupational health (industrial hygiene) and public health. These questions do not imply that commonly proposed strains are intrinsically pathogenic; rather, they highlight the need to evaluate strain persistence, genetic stability, horizontal gene transfer, interaction with soil and water microbiomes, and exposure scenarios across the material life cycle [20,21,22,23]. Existing biosafety and regulatory frameworks were not designed specifically for living construction materials, but they provide useful conceptual references for risk assessment, traceability, contained use, deliberate environmental exposure and precautionary governance [24,25,26,27].

Accordingly, this review integrates molecular, material and biosafety perspectives on bacterial self-healing concrete. Specifically, it examines bacterial stress responses, sporulation and germination mechanisms, MICP pathways, biofilm-associated biomineralization, encapsulation strategies, strain selection criteria and environmental and occupational biosafety considerations. By connecting these dimensions, the review argues that bacterial self-healing concrete should be understood not only as a crack-repair technology, but as a living material system whose responsible development requires coordination between microbiology, molecular biology, materials science, civil engineering, environmental risk assessment, occupational health, and public health.

## 2. Literature Search and Review Approach

### 2.1. Review Design

This article was designed as an integrative narrative review supported by a structured bibliographic search. The objective was not to perform a meta-analysis or a formal systematic review, but to synthesize mechanistic, material and biosafety evidence relevant to bacterial self-healing concrete. The review approach was informed by quality criteria for narrative reviews, particularly the explicit definition of the review aim, transparent description of the literature search, scientific reasoning, appropriate referencing and balanced presentation of evidence [28]. Reporting principles from PRISMA 2020 and PRISMA-ScR were considered only as general references for transparency in evidence identification, but the present work should not be interpreted as a systematic or scoping review [29,30].

### 2.2. Information Sources and Search Period

A structured literature search was conducted in PubMed, Scopus and Web of Science. PubMed was used primarily to identify studies related to bacterial physiology, sporulation, germination, stress responses, biofilms, horizontal gene transfer and biosafety. Scopus and Web of Science were used to retrieve studies from materials science, civil engineering, environmental biotechnology, microbial biomineralization and cementitious biomaterials. The search focused on articles published between January 2019 and August 2026. Foundational studies published before 2019 were included when they provided essential mechanistic background on bacterial alkaline stress, sporulation, germination, calcium carbonate biomineralization, biofilm-associated mineral nucleation or biosafety frameworks.

### 2.3. Search Strategy

Search terms were selected to cover three complementary domains: bacterial self-healing concrete, microbial biomineralization and environmental biosafety. The following terms and combinations were used: “bioconcrete”, “bacterial concrete”, “self-healing concrete”, “microbial self-healing concrete”, “microbially induced calcium carbonate precipitation”, “MICP”, “calcium carbonate precipitation”, “biomineralization”, “*Bacillus subtilis*”, “*Paenibacillus polymyxa*”, “spore-forming bacteria”, “bacterial sporulation”, “spore germination”, “germinant receptors”, “biofilm biomineralization”, “bacterial encapsulation”, “living construction materials”, “engineered living materials”, “horizontal gene transfer”, “*Bacillus* horizontal gene transfer”, “environmental biosafety”, “microbial inoculants”, “soil microbiome”, and “biosafety regulation”. Boolean operators were adapted to the syntax of each database.

### 2.4. Eligibility Criteria

Articles were included when they addressed at least one of the following topics: bacterial self-healing concrete or mortar; MICP in cementitious, geotechnical or environmental applications; calcium carbonate biomineralization by spore-forming bacteria; survival of *Bacillus*, *Paenibacillus* or related taxa under alkaline, osmotic, desiccation or nutrient-limited conditions; molecular regulation of sporulation and germination; biofilm-associated mineral precipitation; bacterial encapsulation systems; engineered living materials; horizontal gene transfer in environmental bacteria; microbial inoculant safety; or regulatory frameworks relevant to microorganisms, environmental exposure or biosafety.

Articles were excluded when they focused exclusively on clinical infections, probiotics, biomedical implants, hospital disinfection, antimicrobial nanoparticles, pharmacological applications or animal studies without conceptual relevance to cementitious biomaterials, environmental biomineralization, bacterial persistence or biosafety. Studies on bacteria outside cementitious matrices were considered only when they provided mechanistic information directly applicable to stress tolerance, sporulation, germination, biomineralization, biofilm formation or genetic exchange.

Clinical case reports were not used to infer infection risk from bioconcrete directly; however, selected reports were considered when necessary to contextualize the documented pathogenic potential of candidate bacterial taxa for the public-health and occupational-risk discussion.

### 2.5. Study Selection and Evidence Prioritization

The final selection prioritized studies with direct relevance to bacterial self-healing concrete, MICP, cementitious materials, microbial survival, encapsulation or environmental implementation. Experimental studies performed in concrete, mortar, cement paste or cementitious composites were prioritized for material-performance statements. Microbiological and molecular studies performed outside cementitious matrices were used to support mechanistic interpretation, particularly when direct evidence in concrete was unavailable. Review articles were used to contextualize the field, identify recurrent limitations and support conceptual integration, but mechanistic claims were preferentially based on primary experimental studies whenever available. Regulatory documents and institutional manuals were used for the biosafety and governance discussion.

### 2.6. Data Extraction and Thematic Synthesis

Relevant information was extracted and organized according to six thematic axes: (i) concrete as a restrictive microbial environment; (ii) bacterial stress responses, sporulation and germination; (iii) MICP pathways and molecular regulation of biomineralization; (iv) biofilms and encapsulation systems as functional interfaces between bacteria and cementitious matrices; (v) bacterial strain selection, including *Bacillus*, *Paenibacillus* and engineered strains; and (vi) environmental and occupational biosafety, horizontal gene transfer, microbiome interactions, public-health considerations and regulatory frameworks. The synthesis emphasized the relationship between biological mechanisms and material-level performance, with particular attention to the transition from bacterial persistence to metabolic reactivation and crack sealing.

### 2.7. Methodological Limitations

This review has limitations inherent to integrative narrative approaches. First, no quantitative meta-analysis was performed because the available studies differ substantially in bacterial species, encapsulation systems, crack widths, curing conditions, nutrient sources, cementitious matrices and outcome measures. Second, the evidence base remains uneven: material-performance studies frequently report crack sealing and mechanical recovery, whereas molecular studies often examine bacterial mechanisms outside concrete-relevant environments. Third, direct evidence on long-term environmental exposure, microbiome effects and biosafety monitoring of bacterial self-healing concrete remains limited. Therefore, mechanistic extrapolations from microbiology, biofilm biology or environmental biotechnology were interpreted cautiously and identified as areas requiring experimental validation under cementitious and field-relevant conditions.

## 3. Thematic Results of the Literature Synthesis

### 3.1. Main Thematic Domains Identified in the Literature

The literature reviewed was organized into thematic domains rather than analyzed quantitatively, due to the heterogeneity of bacterial strains, cementitious matrices, crack widths, curing conditions, encapsulation systems, nutrient sources and outcome measures. The evidence clustered around six main domains: material-level self-healing performance, bacterial persistence under concrete-relevant stress, sporulation and germination mechanisms, MICP pathways and biomineralization regulation, biofilm and encapsulation systems, and environmental biosafety considerations. This thematic organization revealed that bacterial self-healing concrete is most frequently evaluated through crack sealing, permeability reduction and mechanical recovery, whereas the molecular determinants of bacterial survival, latency exit and localized biomineralization remain less consistently integrated into materials-oriented studies. The thematic distribution of the reviewed evidence is summarized in Table 1. This evidence map shows that bacterial self-healing concrete has been most frequently assessed from a material-performance perspective, whereas molecular mechanisms of bacterial persistence, latency exit and environmental biosafety remain less consistently integrated into concrete-oriented studies.

### 3.2. Self-Healing Performance and Limitations in Cementitious Matrices

Experimental studies in mortar and cementitious systems indicate that *Bacillus*-based formulations can contribute to crack sealing and partial recovery of mechanical or durability-related properties. *Bacillus subtilis* has been reported to improve self-healing behavior under different crack widths and curing conditions, supporting its use as a reference organism in bacterial concrete research [7]. Encapsulated *Bacillus subtilis* bio-mortars have also shown potential under chemically restrictive environments, including acidic conditions, suggesting that encapsulation can expand the functional range of bacterial self-healing systems [31]. Additional evidence indicates that *Bacillus subtilis*-instigated calcite precipitation may influence damage progression and ionic transport, linking bacterial mineral precipitation with material durability outcomes [32].

However, the reviewed studies also show that self-healing efficiency is not determined by bacterial presence alone. Crack width, water availability, oxygen diffusion, nutrient source, calcium availability, curing conditions and compatibility between the encapsulation system and cementitious matrix all condition the final performance of the material [7,19,31,32]. Therefore, crack closure should be interpreted as the visible endpoint of a broader sequence involving microbial survival, activation and mineral precipitation under restrictive physicochemical conditions.

### 3.3. Bacterial Survival, Sporulation and Germination Under Concrete-Relevant Stress

Concrete represents a hostile environment for microbial life. High alkalinity, osmotic pressure, desiccation, nutrient limitation and physical confinement may compromise vegetative growth and reduce metabolic activity [8,9]. Under these conditions, bacterial persistence depends on adaptive stress responses and, in spore-forming taxa, on the capacity to enter a latent endospore state. Evidence from *Bacillus subtilis* indicates that environmentally stressed cells may activate physical barriers, stress tolerance mechanisms and viable but nonculturable states, suggesting that bacterial viability in cementitious matrices should not be inferred only from immediate culturability [9].

Sporulation appears as a central survival mechanism for bacterial self-healing concrete. In *Bacillus subtilis*, sporulation is regulated by a complex phosphorelay centered on the master regulator Spo0A, which integrates environmental signals related to nutrient limitation, population status and stress [33]. Endospores provide structural protection against desiccation, nutrient scarcity and prolonged environmental stress, allowing bacteria to persist until crack formation permits the ingress of water and dissolved nutrients [10,11]. This process is relevant for bioconcrete because the functional microorganism must often survive the mixing, setting and service-life phases before contributing to mineral precipitation.

Germination is the transition point between passive persistence and active biomineralization. In *Bacillus subtilis*, germination involves nutrient germinant receptors such as GerA, GerB and GerK, which recognize amino acids, nucleosides and related compounds, triggering downstream events such as dipicolinic acid release, cortex hydrolysis and reactivation of metabolism [34,35]. SpoVA proteins and cortex-lytic enzymes such as CwlJ and SleB are also involved in the structural conversion from dormant spore to vegetative cell [35]. In concrete, water ingress through cracks may provide an initial permissive signal, but efficient germination is likely to depend on the local availability of specific chemical cues. This indicates that the fissure microenvironment, rather than the bulk concrete matrix alone, should be considered a determinant of bacterial self-healing performance.

### 3.4. MICP Pathways and Molecular Regulation of Biomineralization

MICP constitutes the functional mechanism through which bacterial activity is translated into crack sealing. The reviewed literature indicates that calcium carbonate precipitation can occur through ureolytic and non-ureolytic pathways, as well as through heterotrophic metabolism involving organic carbon and calcium sources [36,37]. Ureolytic pathways have been widely explored because they can efficiently increase carbonate availability; however, alternative non-ureolytic or mixed metabolic systems are increasingly relevant due to concerns regarding by-products, sustainability and compatibility with cementitious materials [36,37].

In *Bacillus subtilis*, biomineralization is not merely a passive physicochemical event. A gene cluster involved in calcium carbonate biomineralization has been described, supporting the existence of biological regulation in the precipitation process [13]. Genetic optimization of *Bacillus subtilis* has been shown to improve bacteria-induced calcite precipitation, suggesting that strain-level molecular determinants can influence MICP efficiency [14]. Engineering approaches aimed at CO_2_ sequestration through *Bacillus subtilis* further support the possibility of improving precipitation capacity through metabolic and genetic design [15]. In related spore-forming bacteria, the calcium ATPase YloB has been implicated in calcite precipitation and sporulation, suggesting that calcium transport may represent an additional molecular determinant of biomineralization efficiency [38].

These findings support the need to evaluate MICP as a regulated biological process embedded within a cementitious environment. The precipitation of calcium carbonate depends not only on calcium and carbonate availability, but also on bacterial viability, metabolic activation, ion transport, local pH modulation and the capacity of cells to organize mineral nucleation at the crack interface. The main molecular and functional determinants identified in the literature are summarized in Table 2. These components illustrate that bacterial self-healing performance depends on a coordinated sequence of stress tolerance, sporulation, germination, metabolic reactivation, biofilm formation and calcium carbonate precipitation.

### 3.5. Biofilms and Encapsulation Systems as Functional Interfaces

Biofilm establishment precedes biofilm stabilization and represents a potentially important functional step for localized biomineralization. In *Bacillus subtilis*, biofilm development involves a regulated transition from motile or initially attached cells toward matrix-producing subpopulations. Spo0A-mediated signaling and the SinI-SinR regulatory system contribute to the activation of matrix-associated genes, whereas extracellular polysaccharides and the matrix protein TasA contribute to the structural organization and cohesion of the bacterial community [48,49,50]. In the context of bacterial self-healing concrete, the capacity to establish a biofilm matrix at the crack interface may therefore be as important as its subsequent stability, because such a matrix could promote local concentration of bacterial cells, ions, and mineral nucleation sites. However, these mechanisms have been characterized primarily in microbiological model systems and require direct validation within cementitious crack microenvironments.

Biofilms may act as biological-mineral interfaces in bacterial self-healing concrete. Studies on *Bacillus subtilis* biofilms have shown that extracellular matrix components can contribute to the spatial organization of mineral scaffolds and provide nucleation sites for calcium carbonate precipitation [16,17]. This suggests that biofilms may concentrate bacterial cells, extracellular polymers, ions and mineral precursors at the crack surface, thereby favoring localized precipitation. Elevated calcium concentrations have also been reported to prevent *Bacillus subtilis* biofilm dispersal, stabilizing the matrix architecture [39]. Since cementitious materials are calcium-rich, this mechanism may be relevant for bioconcrete, although direct validation inside concrete matrices remains necessary.

**Figure 1:**
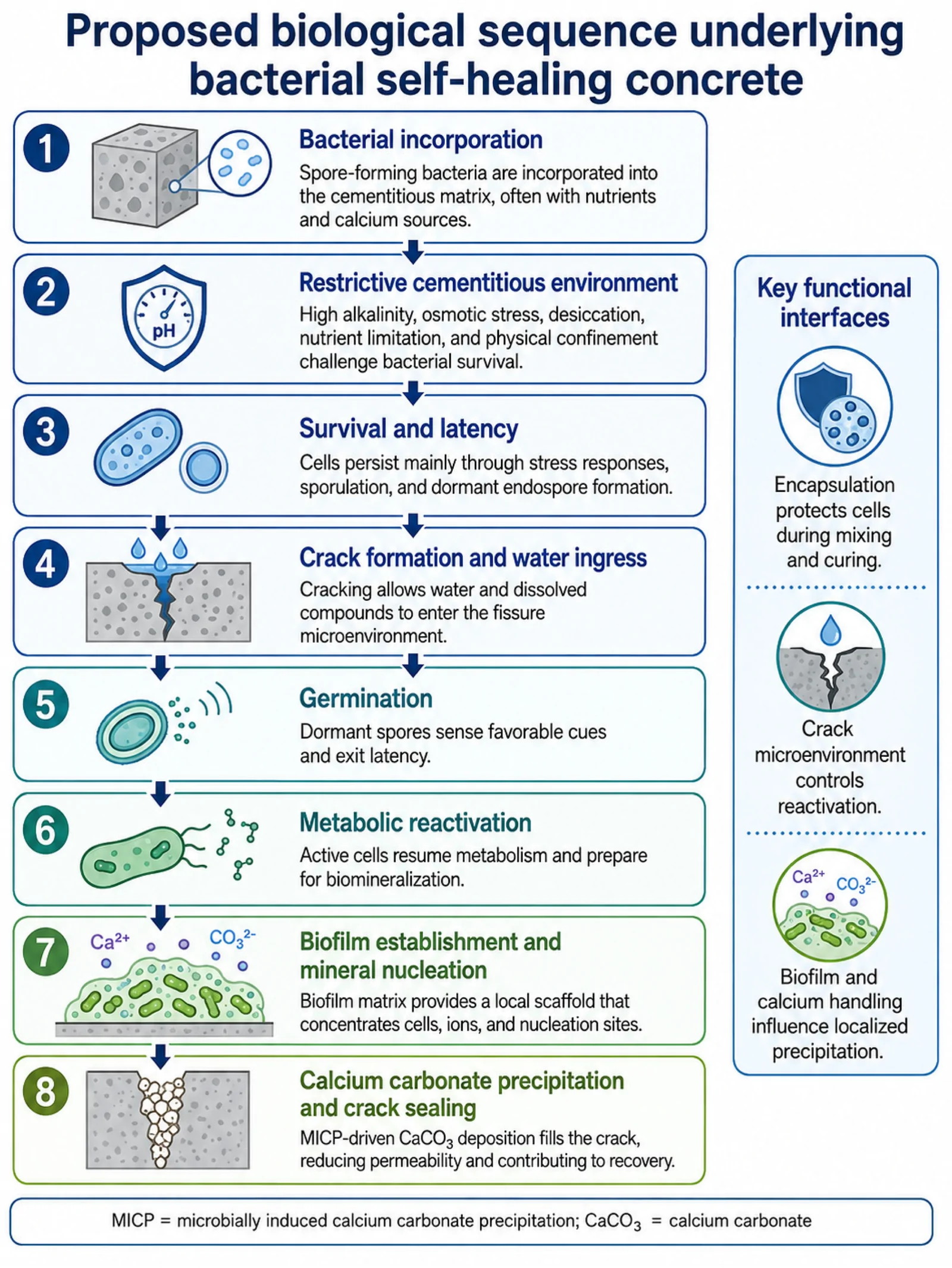
Proposed biological sequence underlying bacterial self-healing concrete: survival, latency, germination, metabolic reactivation, biofilm-associated mineral nucleation and calcium carbonate precipitation. ChatGPT (OpenAI, version 5.5) was used to assist with the graphical rendering of Figure 1, which was conceptually designed and verified by the authors, who take full responsibility for its content.

Encapsulation systems represent a second functional interface between bacterial biology and material performance. Their role is to protect spores or vegetative cells during concrete mixing and curing, preserve viability during latency and enable controlled exposure to water, oxygen and nutrients after cracking. Engineering *Bacillus subtilis* for durable living biocomposite materials demonstrates the potential of combining strain engineering with material design [18]. Studies on calcium carbonate precipitation inside superabsorbent polymers further show that oxygen diffusion, nutrient availability and encapsulant microenvironment can restrict or enable post-release bacterial activity [19]. Therefore, encapsulation should not be treated only as a delivery system, but as a determinant of microbial reactivation and MICP efficiency. The proposed biological sequence underlying bacterial self-healing concrete is illustrated in Figure 1. This model emphasizes that crack sealing is not the result of bacterial incorporation alone, but the endpoint of a coordinated process involving survival, latency, germination, metabolic reactivation, biofilm-associated mineral nucleation and calcium carbonate precipitation.

Available evidence does not identify a single universally optimal encapsulation system for bacterial self-healing concrete. From a functional perspective, an appropriate carrier should protect spores during mixing and high-pH curing, remain mechanically compatible with the cementitious matrix without causing excessive porosity, and permit post-cracking access to water, oxygen, nutrients, and calcium sources required for bacterial reactivation and MICP. Sodium-alginate microencapsulation represents a reproducible, low-temperature option; *Bacillus subtilis* spores at approximately 10^8^ cells/mL have been encapsulated in 2% sodium alginate and subsequently freeze-dried, with the resulting microcapsules showing good integration within mortar and favorable mechanical and water-absorption outcomes [31]. Porous lightweight aggregates can alternatively act as reservoirs for bacterial spores and nutrients; such systems have enabled healing of cracks up to 0.46 mm after water exposure [52]. Silica-gel and polyurethane immobilization have also been investigated, with direct experimental comparison (using *Bacillus sphaericus*) indicating carrier-dependent performance and greater potential of polyurethane for crack repair under the evaluated conditions [53]. Therefore, encapsulant selection should be application-specific and assessed through post-mixing viability, reactivation following water ingress, crack-closure or permeability recovery, and mechanical compatibility with the cementitious matrix.

In practical terms, latency-exit (germination) kinetics can be screened with accessible proxies rather than specialized microbiology: turbidity or optical-density loss upon nutrient addition, colony-forming-unit recovery after simulated cracking [11,35], and onset of CaCO_3_ precipitation tracked by pH rise or calcium depletion [13,14]. These provide engineers with a reproducible readout of bacterial reactivation without requiring full spore-physiology characterization.

### 3.6. Bacterial Strain Selection: Bacillus, Paenibacillus and Engineered Strains

*Bacillus* remains the dominant bacterial model in self-healing concrete due to its capacity for endospore formation, environmental resilience and documented ability to induce calcium carbonate precipitation [7,13,14]. *Bacillus subtilis* is particularly relevant because its sporulation, germination and biofilm biology are well characterized, allowing stronger mechanistic interpretation than is currently possible for many other candidate taxa [16,17,33,34,35].

*Paenibacillus* and related spore-forming bacteria are of biotechnological interest because they share survival traits relevant to restrictive environments, but the evidence directly connecting them to cementitious self-healing remains comparatively limited. Recent findings showing that *Paenibacillus* encodes a membrane-localized Spo0B, unlike the cytosolic Spo0B described in *Bacillus subtilis*, suggest that sporulation initiation may differ between these genera [40]. This difference may have implications for environmental signal integration and latency regulation, but its functional relevance in cementitious matrices remains to be experimentally determined.

Engineered strains offer opportunities to enhance MICP efficiency, stress tolerance or CO_2_ sequestration capacity [14,15,18]. Nevertheless, strain engineering also increases the need for genetic stability assessment, traceability and biosafety evaluation, particularly if the system is intended for open or semi-open environmental deployment. Therefore, bacterial selection should consider not only precipitation capacity, but also sporulation efficiency, germination kinetics, biofilm behavior, compatibility with encapsulation systems and environmental risk profile.

### 3.7. Biosafety, Occupational and Environmental Exposure, and Regulatory Considerations

The reviewed literature indicates that biosafety considerations in bacterial self-healing concrete should be framed from a preventive environmental perspective. For commonly proposed low-risk strains, the main concern is not direct pathogenicity, but the possibility of persistence outside the intended matrix, interaction with native microbiomes, genetic exchange and exposure during production, service life, weathering, demolition or disposal. In *Bacillus* species, natural transformation, biofilm-associated gene transfer, kin discrimination-mediated exchange and mobile genetic elements have been described [41,42,43,44,45]. These mechanisms do not imply an immediate hazard in bioconcrete applications, but they justify evaluating genetic stability and environmental behavior before large-scale implementation.

Environmental conditions may also influence horizontal gene transfer. Soil minerals can modulate bacterial transformation, indicating that the surrounding environment may affect genetic exchange dynamics [23]. Chemical additives or preservatives used in material formulations may also modify rates of mutation or horizontal gene transfer in bacteria [45]. These findings suggest that biosafety assessment should include not only the selected strain, but also the formulation components, encapsulants, additives and environmental context of deployment.

Evidence directly addressing the impact of bacterial self-healing concrete on soil or water microbiomes remains limited. However, studies on microbial inoculants indicate that high-throughput sequencing can support safety assessment, taxonomic verification and compliance monitoring [46]. This approach may be useful for future bioconcrete studies seeking to evaluate whether introduced strains persist, decline or modify local microbial communities after environmental exposure.

Existing regulatory instruments do not specifically address living construction materials. The Cartagena Protocol provides principles for risk assessment and management of living modified organisms [24]. European Directives 2001/18/EC and 2009/41/EC regulate deliberate environmental release and contained use of genetically modified microorganisms, respectively [25,26]. REACH is relevant for chemical components, additives and encapsulating agents, but does not function as the primary framework for regulating viable microorganisms [47]. The WHO Laboratory Biosafety Manual provides a risk-assessment-based approach for biological agents, although it is not specific to environmental deployment in construction materials [27]. Together, these frameworks offer conceptual references for traceability, monitoring and precautionary governance in future bacterial self-healing concrete applications. A life-cycle-oriented biosafety framework is proposed in Table 3. This matrix does not assume direct pathogenicity of the strains used in bacterial self-healing concrete, but identifies stages where environmental exposure, genetic stability, microbial persistence and formulation components should be assessed before large-scale deployment.

To move from a descriptive toward a more operational life-cycle biosafety approach, and for conceptual purposes only, we propose a research-oriented tiered control framework (Table 4). This framework is not intended to constitute a regulatory classification. Tier A comprises well-characterized, non-engineered, low-risk spore-forming strains; Tier B comprises non-engineered environmental isolates for which genomic, toxicological, or persistence-related characterization remains incomplete; and Tier C comprises genetically engineered strains or strains carrying introduced genetic constructs. Monitoring intensity and control measures should increase according to biological uncertainty and the degree of genetic modification.

Monitoring should combine culture-based viable counts with strain-specific molecular methods such as quantitative PCR (qPCR) or digital PCR (dPCR). Where discrimination between viable and inactivated spores is required, propidium-monoazide-based PCR approaches may be considered after appropriate validation for the relevant cementitious or environmental matrix [52]. During demolition and waste-disposal stages, air, dust, concrete debris, and leachate should be sampled. Genetically engineered strains additionally require construct-specific tracking and genetic-stability assessment, consistent with risk-based frameworks for the contained use of genetically modified micro-organisms [26]. Detection of a genetic marker by qPCR or dPCR should be regarded as a surveillance signal rather than sufficient evidence of horizontal gene transfer (HGT); confirmation of HGT requires demonstration that the marker has become associated with a recipient organism, for example through recipient isolation followed by genotyping/sequencing or other sequence-linkage approaches.

From an occupational-health perspective, exposure to viable cells or spores, microbial products, and conventional concrete dust should be considered separately. *Bacillus subtilis* can produce extracellular proteases, including subtilisin (AprE) [54], and occupational exposure to concentrated industrial subtilisins is a recognized cause of respiratory sensitization and enzyme-associated asthma [55]. However, there is currently no direct evidence demonstrating that *B. subtilis*-containing self-healing concrete releases subtilisin at concentrations capable of inducing sensitization during mixing, service life, cutting, or demolition. This hazard should therefore be regarded as plausible but unquantified and likely dependent on the bacterial strain, metabolic state, and material formulation. Future studies could quantify extracellular protease activity and, where aerosolization is plausible, airborne spores and microbial proteins.

Although *B. subtilis* is generally regarded as having low pathogenicity, it should not be considered incapable of causing infection. Rare invasive infections have been reported, particularly in immunocompromised individuals or in the presence of disrupted anatomical barriers, and exceptional cases have also been described in immunocompetent individuals [56]. These clinical observations do not establish an infection risk from bacterial self-healing concrete, but they support a precautionary approach for susceptible workers and high-exposure scenarios.

A distinct occupational issue is exposure to respirable crystalline silica generated during cutting, drilling, grinding, crushing, or demolition of concrete [57]. Evidence from silicosis research indicates that silica-related lung disease can be accompanied by alterations in respiratory microbial communities [58]. However, no direct evidence currently establishes whether simultaneous exposure to spores released from bacterial concrete increases opportunistic-infection risk, adversely modifies the lung microbiome, or produces any beneficial effect. Claims in either direction would therefore be premature. Future field implementation should be accompanied by aerosolization studies, inhalation-toxicology approaches, and, where justified, longitudinal assessment of respiratory microbiome responses [59].

Commercial experience indicates that selected low-risk bacterial self-healing systems have progressed beyond laboratory-scale development. One example is Basilisk Self-Healing Concrete, a TU Delft spin-off [60] that has developed bacteria-based agents for new concrete and repair systems for existing concrete structures. The safety documentation for these products identifies spores of alkaliphilic spore-forming bacilli classified as Group 1 biological agents under Directive 2000/54/EC [61,62], corresponding to biological agents that are unlikely to cause human disease. The Basilisk Healing Agent has also obtained CE certification according to EN 934-2:2009+A1:2012 [62]. However, CE marking for construction products should be interpreted as conformity with the product’s declared performance and applicable harmonized requirements rather than as an absolute demonstration of absence of occupational or public-health hazards. Indeed, the corresponding safety data sheets retain precautionary statements for skin, eye, and respiratory irritation associated with other components of the formulation [62]. Thus, the Basilisk experience provides relevant evidence that low-risk spore-forming bacterial systems can achieve regulated commercial implementation, while still requiring product-, strain-, and exposure-specific risk assessment.

In contrast, evidence for *Paenibacillus* in bacterial self-healing concrete remains considerably less extensive, and the commercial or biosafety evidence available for bacilli used in established products should not be extrapolated directly to other species or strains. Candidate *Paenibacillus* strains should therefore be evaluated at the strain level, considering not only sporulation and biomineralization capacity but also their genetic and secondary-metabolite profiles. For example, a polymyxin synthetase gene cluster has been experimentally identified in *Paenibacillus polymyxa* E681 [63], demonstrating that specific strains can produce biologically active secondary metabolites. Therefore, before large-scale incorporation into bioconcrete, candidate *Paenibacillus* strains should undergo taxonomic and genomic authentication, secondary-metabolite assessment, genetic-stability analysis, and exposure-specific biosafety evaluation.

**Table 3:** Biosafety considerations across the life cycle of bacterial self-healing concrete.

Life-Cycle Phase	Possible Exposure Route	Main Biosafety Issue	Suggested Monitoring or Mitigation Strategy	Regulatory or Conceptual Reference
Strain development and laboratory handling	Laboratory manipulation of viable or engineered microorganisms	Strain identity, genetic stability, containment level	Strain authentication, genome documentation, laboratory risk assessment, contained-use procedures	WHO Laboratory Biosafety Manual; Directive 2009/41/EC [26,27]
Concrete formulation and mixing	Aerosols, accidental release, contact with bacterial carriers	Worker exposure and unintended environmental release during preparation	Use of low-risk strains, encapsulation, personal protective equipment (PPE), batch traceability, and contained preparation	WHO risk assessment principles [27]
Curing and early material setting	Survival inside alkaline matrix; possible release from surface pores	Persistence of viable cells outside intended matrix	Viability assays, leachate testing, surface sampling	Precautionary risk assessment [24,27]
Service life of infrastructure	Water ingress through cracks; runoff after rain or washing	Release or migration of bacteria, spores or DNA fragments	Periodic environmental sampling, qPCR or sequencing-based strain tracking	Cartagena Protocol principles; microbial inoculant monitoring [24,27]
Crack formation and self-healing activation	Local bacterial germination and metabolic reactivation	Localized proliferation, HGT in moist microenvironments	Genetic stability testing, monitoring of mobile genetic elements and resistance markers	HGT literature [23,41,42,43,44,45]
Interaction with soil or water microbiomes	Runoff, leaching, adjacent soil contact	Alteration of native microbial communities	High-throughput sequencing of soil/water microbiomes before and after deployment	Microbial inoculant safety frameworks [27]
Use of additives, nutrients and encapsulants	Chemical leaching or degradation of formulation components	Toxicity, mutation rate changes, HGT modulation	Chemical safety assessment of additives, precursor screening, REACH-based evaluation	REACH; material preservative studies [45,47]
Mixing, cutting and demolition (occupational)	Cement dust, respirable crystalline silica, bacterial spores/cells and potentially microbial proteins	Occupational respiratory exposure; sensitization and opportunistic-infection uncertainty	Personal/area air sampling; respirable-silica monitoring; viable spore counts or strain-specific molecular detection; protease activity when relevant; wet methods, local exhaust/dust suppression and appropriate PPE	Occupational silica-control guidance and Directive 2000/54/EC [61,64]
Demolition and waste disposal	Dust, fragments, runoff, landfill exposure	Environmental dissemination of viable spores or DNA	Waste classification, dust control, viability testing in debris, disposal protocols	Cartagena Protocol principles; WHO risk assessment [24,27]

**Table 4:** Proposed research-oriented tiered control framework for life-cycle biosafety monitoring of bacterial self-healing concrete (proposed by the authors for conceptual purposes; not a regulatory classification).

Life-Cycle Stage	Tier A (Well-Characterized, Non-Engineered)	Tier B (Non-Engineered, Incomplete Characterization)	Tier C (Engineered Strains)	Quantitative Monitoring
Preparation and mixing	Identity, viability, batch traceability	Tier A + extended characterization	Tier B + construct stability and specific containment	CFU, strain-specific qPCR; PMA-PCR when viability is required
Service life/cracking	Follow-up if release occurs	Periodic persistence monitoring	Construct and genetic-stability monitoring	qPCR/dPCR in runoff, leachate or surface samples
Soil and water	Baseline and post-exposure sampling	More frequent follow-up	Strain + construct + potential-recipient tracking	qPCR/dPCR and microbial-community analysis
Demolition and waste	Dust control + spore quantification	Tier A + persistence monitoring in waste	Tier B + construct-specific and HGT tracking	Air/dust/debris: culture, qPCR/dPCR; HGT confirmation by isolation/sequencing

Abbreviations: CFU, colony-forming units; qPCR, quantitative polymerase chain reaction; dPCR, digital polymerase chain reaction; PMA-PCR, propidium monoazide polymerase chain reaction; HGT, horizontal gene transfer.

As summarized in Table 5, the comparison highlights unequal levels of evidence maturity across bacterial systems. *Bacillus subtilis* is supported by direct crack-healing studies using defined crack widths and by encapsulation experiments, whereas emerging evidence for *Paenibacillus polymyxa* indicates promising concrete-performance outcomes but remains less extensive and less standardized. Genetically engineered strains offer opportunities to enhance molecular pathways associated with MICP; however, improved mineral precipitation or CO_2_ sequestration cannot be assumed to translate directly into superior crack-healing performance within cementitious matrices. Reliable cross-strain benchmarking will therefore require a minimum common reporting set including initial and final crack width, bacterial concentration and strain identity, incorporation or encapsulation method, curing regime, nutrient and calcium sources, post-mixing viability, germination/reactivation capacity, CaCO_3_ characterization, permeability recovery, mechanical recovery, and follow-up duration.

## 4. Discussion: From Molecular Mechanisms to Responsible Implementation

The evidence synthesized in this review supports a conceptual shift in the interpretation of bacterial self-healing concrete. Rather than being understood only as a cementitious material capable of precipitating calcium carbonate, bioconcrete should be approached as a living or bioactive material system in which biological persistence, metabolic reactivation, mineral nucleation and material compatibility must occur in a coordinated sequence. As summarized in Table 1 and Figure 1, crack sealing represents the visible endpoint of this process, but its success depends on upstream biological events that are often underrepresented in material-performance studies.

A central implication of this review is that the efficacy of bacterial self-healing concrete cannot be predicted solely from the capacity of a strain to induce calcium carbonate precipitation under permissive laboratory conditions. The cementitious matrix imposes high alkalinity, osmotic stress, desiccation, nutrient limitation and physical confinement, all of which may compromise vegetative growth and delay metabolic recovery [8,9,33,34]. Therefore, bacterial survival strategies, especially sporulation and latency maintenance, should be considered functional determinants of self-healing performance. In this context, sporulation is not merely a survival trait, but a design-relevant biological process that allows bacteria to persist during mixing, curing and service life until crack formation creates a more permissive microenvironment [10,11,33].

Germination emerges as a particularly important but insufficiently evaluated transition point. The activation of dormant spores requires specific molecular and environmental signals, including nutrient germinants, water availability and downstream structural remodeling involving germinant receptors, dipicolinic acid release and cortex degradation [34,35]. For this reason, the crack microenvironment should be treated as a critical functional niche. Its local pH, moisture, oxygen availability, calcium concentration and supply of amino acids or nucleosides may determine whether spores remain dormant or become metabolically active. This perspective suggests that future bioconcrete formulations should not only report bacterial viability before incorporation, but should also evaluate latency-exit kinetics and metabolic reactivation under simulated crack conditions.

The molecular regulation of biomineralization also deserves greater attention. Although MICP is frequently presented as a physicochemical precipitation process, evidence from *Bacillus subtilis* and related spore-forming bacteria indicates that calcium carbonate precipitation may involve specific genetic determinants, metabolic regulation and calcium transport mechanisms [13,14,15,38,65]. These findings support the interpretation of MICP as a biologically regulated phenomenon embedded within the chemical environment of concrete. As shown in Table 2, molecular components involved in stress response, sporulation, germination, calcium handling and biofilm formation may collectively influence the final efficiency of CaCO_3_ precipitation.

Biofilms and encapsulation systems represent two key interfaces between microbial physiology and material performance. Biofilms may spatially organize bacterial cells, extracellular polymers and mineral nucleation sites at the crack surface, thereby favoring localized precipitation [16,17,39]. Encapsulation, in turn, protects bacteria during the early phases of concrete preparation and modulates their exposure to water, oxygen, nutrients and calcium sources after cracking [18,19]. This means that encapsulants should not be treated as passive carriers, but as microenvironmental regulators of bacterial reactivation and MICP efficiency. The interaction between encapsulation architecture, bacterial germination and crack chemistry is therefore a priority area for future experimental work.

From a materials science perspective, current evidence supports the potential of *Bacillus*-based systems to improve crack sealing, reduce permeability and contribute to mechanical or durability-related recovery in mortar and cementitious systems [7,31,32,37]. However, comparison across studies remains difficult because of differences in bacterial strains, crack widths, curing regimes, nutrient sources, encapsulation materials, calcium precursors and outcome measures. This heterogeneity limits the ability to establish generalizable performance thresholds. Future studies should adopt more standardized reporting criteria, including initial and final crack width, curing conditions, bacterial concentration, encapsulation method, precursor composition, viability after mixing, germination capacity after cracking, CaCO_3_ polymorph characterization and durability-related outcomes.

The selection of bacterial strains should therefore move beyond a simple screening for precipitation capacity. *Bacillus subtilis* remains a useful reference model because its sporulation, germination, biofilm biology and genetic regulation are comparatively well characterized [13,14,16,17,33,34,35]. *Paenibacillus* and other spore-forming bacteria may offer additional biotechnological possibilities, but their performance under cementitious conditions remains less clearly established. Findings suggesting differences in sporulation signaling, such as membrane-localized Spo0B in *Paenibacillus*, indicate that genus-specific regulatory mechanisms may exist [40]. Nevertheless, their functional relevance for bioconcrete requires direct validation in cementitious matrices. For engineered strains, improvements in MICP efficiency or CO_2_ sequestration capacity must be balanced with genetic stability, traceability and biosafety requirements [14,15,18].

The biosafety dimension should be understood as complementary to technological development, not as an argument against bacterial self-healing concrete. For low-risk, non-pathogenic strains, the main concern is not direct pathogenicity, but environmental persistence, genetic stability, interaction with native microbiomes and possible horizontal gene transfer under specific exposure scenarios [23,41,42,43,44,45]. As presented in Table 3, biosafety assessment should cover the complete material life cycle, including strain development, laboratory handling, formulation, construction, curing, service life, crack activation, runoff, environmental interface, demolition and waste disposal. This life-cycle approach is especially relevant because living construction materials are not yet directly addressed by most existing regulatory frameworks.

Current biosafety and regulatory instruments provide useful conceptual references, although they are not specifically designed for bacterial self-healing concrete. The Cartagena Protocol and European directives on genetically modified microorganisms offer principles related to risk assessment, traceability, contained use and deliberate environmental release [24,25,26]. REACH is relevant for chemical additives, nutrients, precursors and encapsulating agents, but does not regulate viable microorganisms as such [47]. The WHO Laboratory Biosafety Manual provides a general risk-assessment framework for biological agents, although it is oriented primarily toward laboratory biosafety rather than environmental deployment in construction materials [27]. Therefore, future governance of bioconcrete will likely require adapted protocols integrating material safety, microbial ecology, environmental monitoring and public health criteria.

This review also reveals several limitations in the current evidence base. First, many studies focus on short-term crack sealing or mechanical recovery, whereas long-term bacterial viability and field performance remain less frequently assessed. Second, molecular biology studies provide important mechanistic insights but are often performed outside concrete-relevant environments, requiring cautious extrapolation. Third, the interaction between bacterial strains, encapsulation systems and cementitious chemistry is still insufficiently standardized. Fourth, direct evidence on environmental release, soil and water microbiome effects, and long-term biosafety monitoring remains scarce. These limitations do not weaken the relevance of bacterial self-healing concrete but indicate that its development should proceed through integrated experimental designs.

Future research should therefore combine mechanical testing, microbiological viability assays, germination studies, molecular profiling, biomineralization analysis, microscopy, sequencing-based monitoring and environmental risk assessment. Particularly important priorities include: evaluating spore survival and germination under simulated crack microenvironments; comparing *Bacillus*, *Paenibacillus* and other spore-forming taxa under standardized cementitious conditions; characterizing biofilm-associated mineral nucleation inside cracks; optimizing encapsulation systems as biologically active microenvironments; and developing life-cycle biosafety protocols for field-scale applications. Only by integrating these dimensions can bacterial self-healing concrete advance from promising laboratory technology toward responsible, durable and environmentally compatible implementation.

## 5. Conclusions and Future Directions

Bacterial self-healing concrete should be understood as a living or bioactive material system rather than as a passive cementitious matrix containing calcium carbonate-producing bacteria. Its effectiveness depends on the continuity between bacterial survival, sporulation, germination, metabolic reactivation, biofilm-associated mineral nucleation and MICP-mediated crack sealing. Therefore, the biological performance of the selected microorganism is inseparable from the mechanical and durability performance of the final material.

Future development of this technology requires strain selection criteria that integrate alkaline and osmotic stress tolerance, genetic stability, sporulation efficiency, germination kinetics, biomineralization capacity, biofilm behavior and compatibility with encapsulation systems. At the material level, future studies should standardize the reporting of crack width, curing conditions, bacterial concentration, encapsulation method, nutrient and calcium sources, post-mixing viability, CaCO_3_ characterization, permeability reduction, mechanical recovery and long-term durability.

Responsible large-scale implementation also requires environmental biosafety, occupational health, and public-health assessment from the early design stages. Priority areas include life-cycle exposure analysis, monitoring of soil and water microbiomes, horizontal gene transfer assessment, strain traceability, evaluation of additives and encapsulants, and adaptation of existing biosafety frameworks to living construction materials.

Overall, bacterial self-healing concrete represents a promising strategy for more durable and sustainable infrastructure, but its development should proceed through integrated evaluation of microbial viability, molecular regulation, material performance and environmental safety. A transdisciplinary approach linking microbiology, molecular biology, materials science, civil engineering, environmental risk assessment, occupational health, and public health will be essential for translating this technology from experimental systems to responsible field applications.

## Figures and Tables

**Table 1:** Thematic evidence map of bacterial self-healing concrete.

Thematic Domain	Main Evidence Identified	Representative References	Current Limitation	Relevance for Bioconcrete Design
Material-level self-healing performance	*Bacillus subtilis*-based systems can contribute to crack sealing, mechanical recovery and durability-related improvements in mortar or cementitious matrices.	[7,31,32]	Heterogeneous crack widths, curing conditions, formulations and outcome measures limit direct comparison.	Defines the visible endpoint of self-healing: crack closure, permeability reduction and recovery of material performance.
Concrete as a restrictive microbial environment	High alkalinity, osmotic stress, desiccation, nutrient limitation and physical confinement can compromise bacterial viability and metabolic activity.	[8,9,33]	Most mechanistic stress studies are not performed directly inside concrete.	Supports the need to evaluate bacterial survival and not only mineral precipitation.
Sporulation and germination	Endospore formation enables long-term persistence; germination requires specific molecular triggers and structural remodeling.	[10,11,34,35]	Germination kinetics are rarely tested under real crack microenvironments.	Determines whether dormant spores can reactivate after fissure formation.
MICP and molecular biomineralization	Calcium carbonate precipitation involves metabolic pathways, genetic determinants, calcium handling and local physicochemical modulation.	[13,14,15,36,37,38]	MICP is often interpreted chemically, with limited integration of molecular regulation.	Links bacterial metabolism with CaCO_3_ precipitation efficiency and crack sealing.
Biofilms and encapsulation systems	Biofilms may provide mineral nucleation scaffolds; encapsulation protects spores and modulates post-crack access to water, oxygen and nutrients.	[16,17,18,19,39]	Direct evidence inside cementitious cracks remains limited.	Connects microbial physiology with spatially localized biomineralization.
Strain selection	*Bacillus* remains the dominant model; *Paenibacillus* is promising but less validated in cementitious systems.	[7,13,14,40]	Comparative studies under standardized concrete conditions are scarce.	Guides rational selection based on sporulation, germination, MICP capacity and genetic stability.
Biosafety, environmental and occupational exposure	Horizontal gene transfer (HGT), persistence, microbiome interaction and lack of specific regulation require preventive assessment.	[23,24,25,26,27,41,42,43,44,45,46,47]	Few studies evaluate long-term environmental and occupational exposure associated with bacterial self-healing concrete.	Supports traceability, environmental and occupational exposure assessment, monitoring, and risk governance before large-scale implementation.

**Table 2:** Molecular and functional determinants relevant to bacterial self-healing concrete.

Biological Process	Component or Determinant	Main Function	Organism/Context	Relevance to Bioconcrete	Refs.
Alkaline stress response	Ionic homeostasis and stress response networks	Preserves membrane function, protein stability and cellular integrity under high pH.	Environmental bacteria; *Bacillus* spp.	Supports survival in alkaline cementitious matrices.	[8,9,33,34]
Sporulation initiation	Spo0A phosphorelay	Integrates stress, nutrient limitation and population signals to initiate sporulation.	*Bacillus subtilis*	Enables entry into a resistant dormant state before or during concrete service life.	[33]
Sporulation regulation	Spo0B localization	Participates in phosphorelay signaling; membrane localization may differ in *Paenibacillus*.	*Paenibacillus* spp.	Suggests possible genus-specific sporulation responses under environmental stress.	[40]
Germinant recognition	GerA, GerB, GerK receptors	Detects nutrient germinants such as amino acids, nucleosides or related compounds.	*Bacillus subtilis* spores	Determines whether spores can exit latency after water and nutrients enter cracks.	[34,35]
Spore core rehydration	SpoVA proteins	Participates in dipicolinic acid release and early germination events.	*Bacillus subtilis* spores	Supports transition from dormant spore to metabolically active cell.	[35]
Cortex degradation	CwlJ and SleB	Degrades spore cortex peptidoglycan during germination.	*Bacillus subtilis* spores	Allows spore expansion and vegetative recovery after crack activation.	[35]
Biomineralization regulation	Calcium carbonate biomineralization gene cluster	Contributes to biologically regulated CaCO_3_ precipitation.	*Bacillus subtilis*	Indicates that MICP is not merely passive chemical precipitation.	[13]
Enhanced calcite precipitation	Genetic optimization of MICP-related pathways	Increases bacteria-induced calcite precipitation.	Engineered *Bacillus subtilis*	Supports strain improvement as a strategy to enhance self-healing efficiency.	[13,14]
Calcium transport	YloB calcium ATPase	Participates in calcium handling, calcite precipitation and sporulation.	*Solibacillus silvestris*	Suggests calcium transport may influence biomineralization efficiency in spore-forming bacilli.	[38]
Biofilm establishment	Spo0A; SinI-SinR regulatory system	Regulates the transition toward matrix-producing cells and supports early biofilm development	*Bacillus subtilis*	May determine whether an organized microbial interface becomes established at the crack surface before mineral nucleation	[48,49]
Biofilm matrix formation	Extracellular polysaccharides and TasA	Provides structural organization and cohesion to the bacterial community	*Bacillus subtilis* biofilms	May promote local cell retention and provide a functional scaffold for subsequent biomineralization	[48,50]
Biofilm stability	Calcium-mediated biofilm retention	Prevents biofilm dispersal and stabilizes matrix architecture	*Bacillus subtilis* biofilms	May favor persistent biomineralizing biofilms at crack interfaces	[39]
Mineral nucleation	Biofilm extracellular matrix	Provides scaffold and nucleation sites for mineral deposition.	*Bacillus subtilis* biofilms	Supports localized CaCO_3_ precipitation within or near cracks.	[16,17]
Encapsulation interface	Water-responsive protective carriers: sodium alginate, polyurethane or porous lightweight reservoirs	Protects spores during mixing and curing while enabling post-crack access to water, oxygen and nutrients	Encapsulated aerobic bacteria	Carrier selection should balance bacterial viability, activation after cracking and mechanical compatibility with the cementitious matrix	[31,51,52]

**Table 5:** Comparative empirical evidence and reporting gaps among bacterial systems relevant to self-healing concrete.

Scheme	Strain	Strain Type	Bacterial Loading/Incorporation	Carrier/Delivery	Crack/Experimental Conditions	Main Outcome	Comparative Limitation
Yamasamit et al., 2023 [7]	*B. subtilis*	Non-engineered	10^8^ CFU/mL	Direct crack treatment; 2% alginate microencapsulation in parallel bio-mortar	0.3, 0.5 and 1.0 mm; 7–28 d	28 d healing ratios: 77.78%, 76.67% and 63.33%, respectively	Surface treatment not directly equivalent to embedded systems
Rivas-Torres et al., 2026 [49]	*B. subtilis* vs. *P. polymyxa*	Non-engineered	Bacterial solutions; 10%, 15%, 20% water replacement; injection	Bacterial solution	Type IP concrete; 7, 14, 21, 28 d	Up to 335.71 kg/cm^2^ (+59.9% vs. standard design); *P. polymyxa* showed higher performance than *B. subtilis* under several of the evaluated conditions	Variables not equivalent to standardized crack-width/encapsulation protocols
Gilmour et al., 2024 [15]	Engineered *B. subtilis*	Genetically engineered	Recombinant carbonic-anhydrase expression	Not a crack-encapsulated system	MICP/CO_2_-sequestration; no concrete crack test	CO_2_ decreased from 3800 to 820 ppm; calcite and vaterite produced	Demonstrates engineered MICP potential but not concrete crack-healing

Abbreviations: CFU, colony-forming units.

## Data Availability

No new data were created or analyzed in this study. Data sharing is not applicable to this article.
